# (Ir)reversibility of structural and functional brain alterations in severe anorexia

**DOI:** 10.1192/j.eurpsy.2024.36

**Published:** 2024-08-27

**Authors:** L.-K. Kaufmann

**Affiliations:** Radboud University, Donders Institute for Brain, Cognition and Behaviour, Nijmegen, Netherlands

## Abstract

**Abstract:**

Anorexia nervosa is characterized by profound structural and functional brain alterations, particularly during the phase of acute underweight. Understanding the reversibility of these changes upon weight normalization is an important question in the pursuit of recovery and relapse prevention. This talk shares findings from recent neuroimaging studies, focussing on the dynamic processes of brain recovery observed during and after inpatient treatment in individuals with severe anorexia nervosa.

**Disclosure of Interest:**

None Declared

